# Self-Adapting Foot Orthosis Inlay Facilitates Handling and Reduces Plantar Pressure Compared to Vacuum-Based Technology

**DOI:** 10.3390/jcm14103384

**Published:** 2025-05-13

**Authors:** Alexander Milstrey, Carolin Horst, Stella Gartung, Ann-Sophie Weigel, Richard Stange, Sabine Ochman

**Affiliations:** 1 Department of Trauma-, Hand- and Reconstructive Surgery, University Hospital Muenster, 48149 Muenster, Germanystella.gartung@ukmuenster.de (S.G.); annsophie.weigel@ukmuenster.de (A.-S.W.); sabine.ochman@ukmuenster.de (S.O.); 2 Department of Regenerative Musculoskeletal Medicine, Institute of Musculoskeletal Medicine (IMM), University Hospital Muenster, 48149 Muenster, Germany; richard.stange@ukmuenster.de

**Keywords:** orthosis, foot, peak plantar pressure, VACOpedes, insole

## Abstract

**Background/Objectives**: Orthoses are commonly used in the treatment of various foot and ankle injuries and deformities. An effective technology in foot orthoses is a vacuum system to improve the fit and function of the orthosis. Recently, a new technology was designed to facilitate the wearing of the foot orthoses while maintaining function without the need for vacuum suction. **Methods**: A plantar dynamic pressure distribution measurement was carried out in 25 healthy subjects (13 w/12 m, age 23–58 y) using capacitive measuring insoles in two differently designed inlays within the VACOpedes^®^ orthosis (Group A: vacuum inlay vs. Group B: XELGO^®^ inlay) and a regular off-the-shelf shoe (Group C, OTS). The peak plantar pressure, mean plantar pressure and maximum force were analyzed in the entire foot and in individual regions of the medial and lateral forefoot, the midfoot and the hindfoot. Finally, the wearing comfort was compared using a visual analog scale from 1 to 10 (highest comfort). **Results**: The peak pressure of both inlays was significantly lower than in the OTS shoe (A: 230.6 ± 44.6 kPa, B: 218.0 ± 49.7 kPa, C: 278.6 ± 50.5 kPa; *p* < 0.001). In a sub-analysis of the different regions, the XELGO^®^ inlay significantly reduced plantar pressure in the medial forefoot compared to the vacuum orthosis (A: 181.7 ± 45.7 kPa, B: 158.6 ± 51.7 kPa, *p* < 0.002). The wearing comfort was significantly higher with the XELGO^®^ inlay compared to the vacuum inlay (A: 5.68/10, B: 7.24/10; *p* < 0.001). **Conclusions**: The VACOpedes^®^ orthosis with a new XELGO^®^ inlay showed at least equivalent relief in all pressure distribution measurements analyzed and greater relief in the forefoot area than the VACOpedes^®^ orthosis with a vacuum inlay, as well as increased wearing comfort.

## 1. Introduction

Orthoses are commonly used in foot and ankle surgery to limit plantar loading, reduce pain, and restrict movement in the treatment of various injuries or deformities, and the application time is reduced compared to a plaster cast. Further, the orthoses can be taken off and put back on by the patient, which enhances personal hygiene. On the other hand, a previous study has shown that the use of orthoses reduces soft tissue bruising, improves immobilization and optimizes off-loading, resulting in a faster return to work [1]. In addition, some foot orthoses are designed to allow a protected ankle range of motion or full weight bearing in the hindfoot with simultaneous relief of the forefoot [2,3]. As a result, over the past few decades, foot orthoses have become standard of care in the treatment algorithm for various traumatic and degenerative foot and ankle pathologies.

One of the main goals of foot orthoses is the reduction of plantar pressure and load applied to the forefoot and hindfoot. In general, plantar pressure depends on different factors, such as the patient´s age, gender, weight and walking speed [4,5,6,7]. Depending on the type of orthosis, plantar pressure can be significantly reduced by the shape and fit of the inlay within the orthosis. These factors are important in increasing wearing comfort and are therefore essential for patient acceptance and clinical outcome [8,9]. A current concept to improve the fit of a foot orthosis and thereby reduce the peak plantar pressure is the use of a vacuum-based inlay. This inlay is filled with polystyrene beads, which adapt to the anatomical structure of the foot and bond reversibly under vacuum [10]. However, there is evidence that the vacuum fit is highly dependent on accurate application by the patient. Especially in geriatric patients, not all patients are able to apply the vacuum orthosis correctly, resulting in compromised function and comfort [11].

Therefore, a novel technology in foot orthoses has been developed to facilitate application and maintain function. This technology—XELGO^®^ (OPED GmbH, Valley, Germany)—mainly relates to the inlay, which consists of pads filled with polystyrene beads that are moistened with a high-viscosity liquid. The filling adapts itself to the anatomy and the beads combine when the orthosis is loaded. This effect is reversible at any time by kneading the pads and flattening the filling. Without the need for a vacuum pump, the new inlay allows for simple handling of the orthosis.

Hence, the present study aimed to evaluate the effect of a foot orthosis with two different inlay technologies: the vacuum technology and the new self-adapting XELGO^®^ technology on peak plantar pressure, mean plantar pressure and maximum force reduction and wearing comfort. We hypothesized that the new inlay technology would reduce the plantar pressure in the same way as the vacuum inlay while being more comfortable to wear and easier to handle.

## 2. Materials and Methods

### 2.1. Subjects

The study was conducted in 25 healthy Caucasian subjects (13 female, 12 male) in Germany in March 2022. The median age was 31 (range, 23 to 58) years. The median BMI was 25 (range, 19–32) kg/m^2^. All subjects gave informed consent to participate in the study. The inclusion criteria were healthy people aged over 18. Exclusion criteria were a history of lower leg injury, any impairment of gait or foot function (e.g., longitudinal or transverse flatfoot, hallux valgus) or neurological disorder. Prior to inclusion in the study, a specialized foot and ankle surgeon clinically assessed both feet of all subjects for intact integument, alignment, and regular gait pattern. The study was approved by the Ethics Committee of the Medical Council of Westfalen-Lippe, Germany (approval number 2022-036-f-S, approval date 1 March 2022) and was conducted in accordance with the Declaration of Helsinki.

### 2.2. Study Design

**Figure 1:**
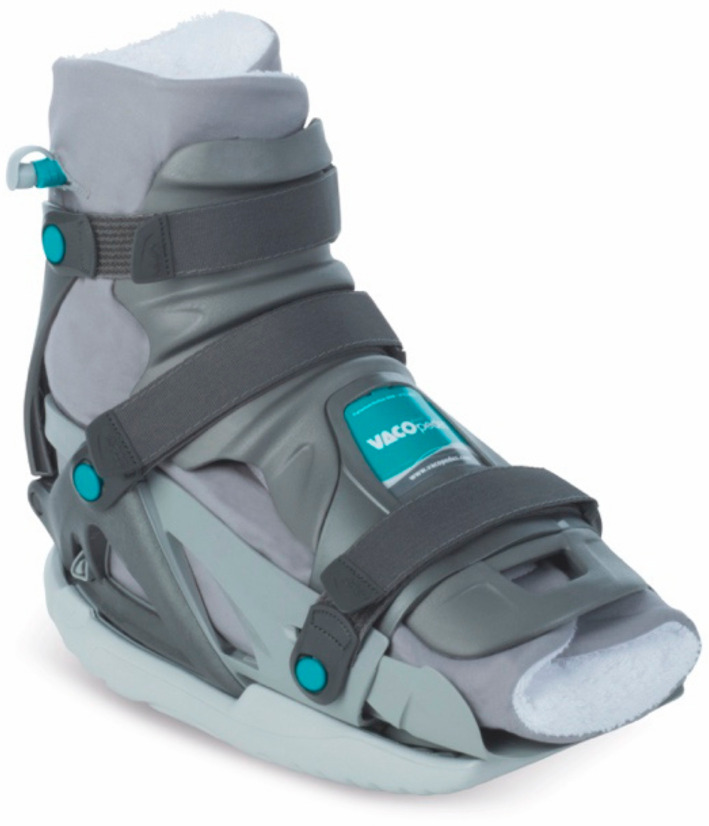
Exemplary VACOpedes^®^ orthosis with vacuum inlay.

**Figure 2:**
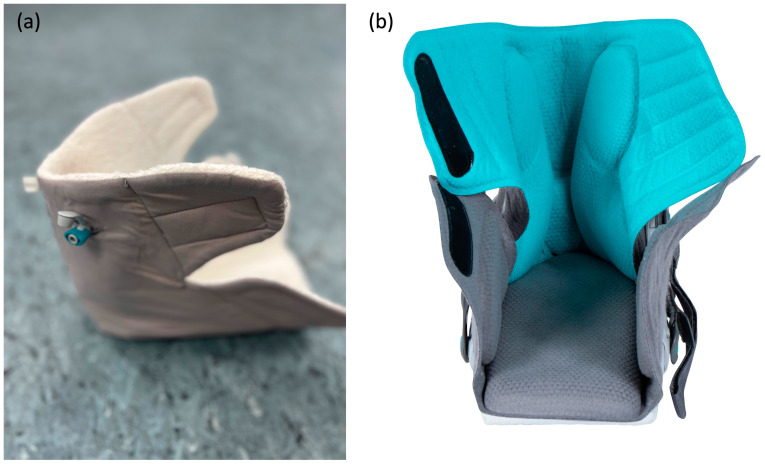
Exemplary insoles of both groups. (**a**) VACOpedes^®^ orthosis. (**b**) XELGO^®^ orthosis with Pedar-X^®^ insole.

This observational cross-sectional study investigated the unloading effect of two different inlay technologies in the VACOpedes^®^ (OPED GmbH, Valley, Germany) foot orthosis (see Figure 1 and Figure 2). This orthosis immobilizes the foot and provides functional treatment for forefoot and midfoot injuries, as well as other metatarsal fractures (I–IV), Hallux valgus/rigidus or arthrodesis of the toe joint. The outer shell consists of a thermoplastic grid shell, it has a rocker sole and a flexible front covers the ankle and foot to fix the foot in a defined position.

To stabilize the foot within the orthosis and optimize pressure distribution, the inlay adapts to the individual foot anatomy. Since the market launch of the VACOpedes^®^ orthosis, the inlay has been based on vacuum technology, where the polystyrene beads are loose under normal conditions and bind under vacuum. Since the development of XELGO^®^, the reversible binding of the polystyrene beads has been based on a highly viscous liquid with which the beads are moistened.

**Figure 3:**
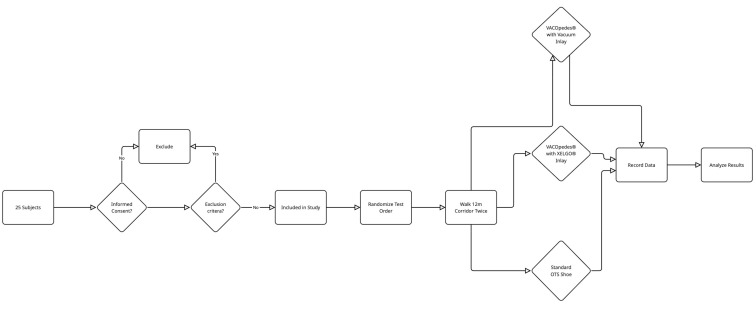
Graphic flow diagram for all subjects throughout the study.

All participants had to walk a 12 m corridor twice under three different conditions: (1) VACOpedes^®^ orthosis with a vacuum inlay, (2) VACOpedes^®^ orthosis with a XELGO^®^ inlay and (3) standard off-the-shelf shoe (OTS, Puma Street cat). Pedobarography was performed using capacitive sensor insoles (Pedar X^®^, Novel GmbH, Munich, Germany) with 99 sensors. The order in which the different shoe conditions were tested was randomized (see Figure 3). Previous studies have shown good reliability of this system [12,13]. The peak plantar pressure, the maximum force and the mean pressure were analyzed by separating the contact area into hindfoot, midfoot and forefoot. Peak pressure was defined as the maximum load under the foot in kPa. The maximum force was defined as the highest vertical force measured by the capacitive sensors in Newtons. Mean pressure was defined as the average pressure exerted on the plantar surface of the foot. The forefoot area was further subdivided into the medial and lateral forefoot area. In addition, a barefoot measurement with a plantar pressure plate (emed, Novel GmbH, Munich, Germany) was performed to assess individual physiological foot geometry and load. The walking speed was controlled with light gates (DLS-LA Timing System, AF Sport, Wesel, Germany). The speed was allowed to vary between individuals but had to remain constant between the different shoe conditions for each subject (mean speed: 4.7 km/h ± 0.5 km/h). The wearing comfort of the VACOpedes^®^ and XELGO^®^ orthoses was analyzed using a visual analogue scale ranging from 1 (no comfort) to 10 (maximum comfort).

### 2.3. Statistics

A priori power analysis was performed using G*Power (version 3.1.9). Based on means and standard deviations from previous biomechanical studies testing different foot orthoses [14], it was determined that a sample size of 23 would be able to detect changes in a peak plantar pressure of 39 kPa (with a standard deviation of 54 kPa) with 95% power at the *p* < 0.05 significance level. Results are presented as mean values for each group with standard error of the mean (SEM). All data were tested for normal distribution with a Shapiro–Wilk test. Since all data were normally distributed, they were analyzed by paired *t*-test, followed by Welch correction. In all cases, significance was set at *p* < 0.05. All statistics were performed using SPSS Statistics (V27.0, IBM, Armonk, NY, USA).

## 3. Results

The parameters were analyzed by separating the contact area into hindfoot, midfoot and forefoot.

**Figure 4:**
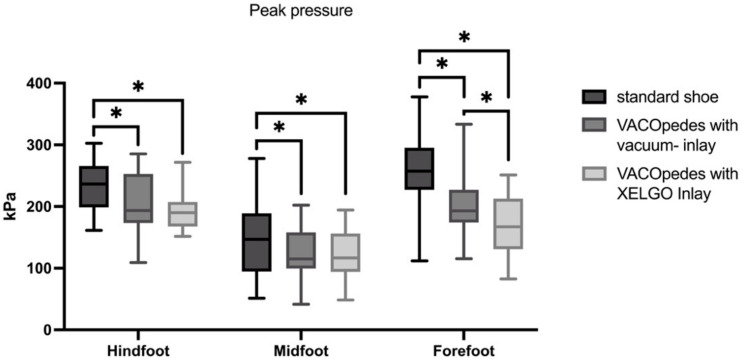
Peak pressure in kiloPascal (kPa) for each group, separated into hindfoot, midfoot and forefoot. Data are presented in box plots, with the median of each group in the center line, the box drawn from the first to the third quartile and whiskers representing the minimum and maximum of each group. A Shapiro–Wilk test confirmed that all data were normally distributed. Significant differences are marked with * (*p* < 0.05).

There was a significant decrease in peak pressure, maximum force and mean pressure in the hindfoot using both inlays compared to the OTS; however, both inlays did not significantly differ from each other (see Figure 4 and Table 1). In the midfoot, both orthoses reduced peak plantar pressure compared to the OTS, with no differences between the two inlays (see Figure 4 and Table 1).

**Figure 5:**
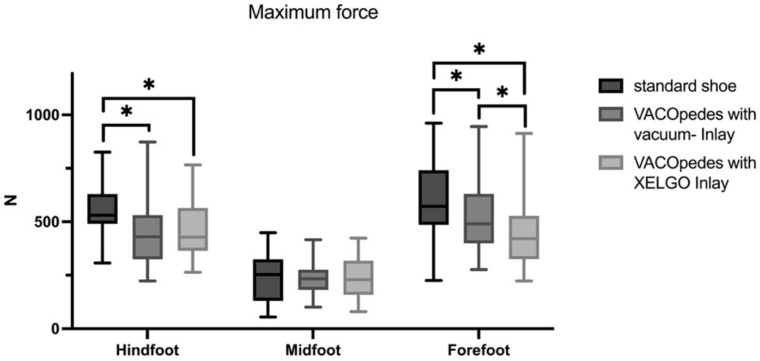
Maximum force in Newton (N) for each group, separated into hindfoot, midfoot and forefoot. Data are presented in box plots, with the median of each group in the center line, the box drawn from the first to the third quartile and whiskers representing the minimum and maximum of each group. A Shapiro–Wilk test confirmed that all data were normally distributed. Significant differences are marked with * (*p* < 0.05).

**Figure 6:**
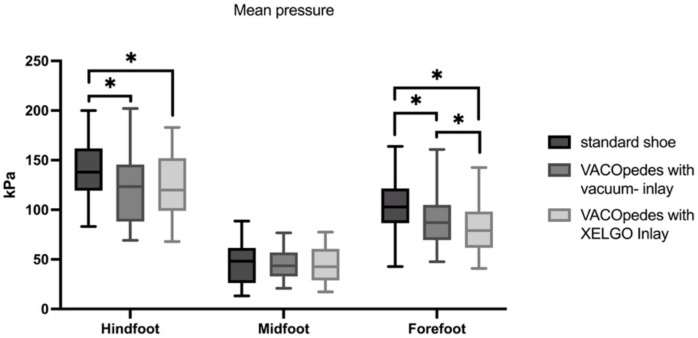
Mean Pressure in kiloPascal (kPa) for each group, separated into hindfoot, midfoot and forefoot. Data are presented in box plots, with the median of each group in the center line, the box drawn from the first to the third quartile and whiskers representing the minimum and maximum of each group. A Shapiro–Wilk test confirmed that all data were normally distributed. Significant differences are marked with * (*p* < 0.05).

Maximum force and mean plantar pressure showed no significant differences in the midfoot (see Figure 5 and Figure 6). Both inlays of the VACOpedes^®^ orthosis significantly decreased peak plantar pressure, maximum force and mean plantar pressure in the forefoot compared to the OTS (Figure 4, Figure 5 and Figure 6 and Table 1). Additionally, the XELGO^®^ inlay reduces forefoot peak pressure (*p* < 0.001), maximum force (*p* < 0.001) and mean pressure (*p* = 0.002) significantly compared to the vacuum inlay (Figure 4, Figure 5 and Figure 6).

**Figure 7:**
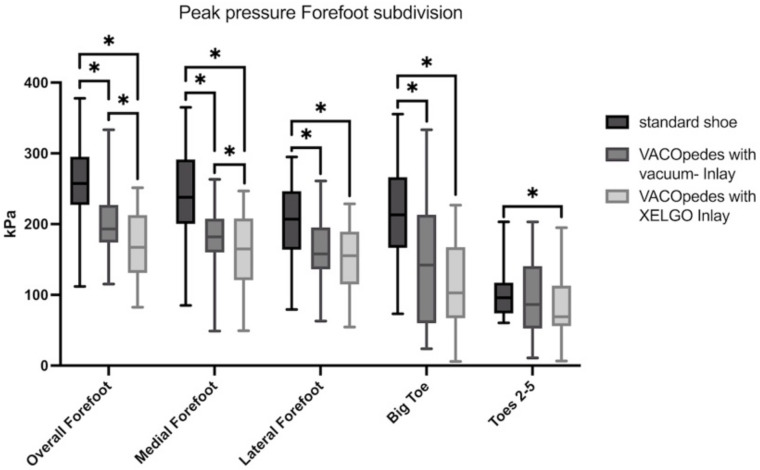
Peak pressure of the forefoot in kiloPascal (kPa) for each group, separated into overall, medial forefoot, lateral forefoot, big toe and toes 2–5. Data are presented in box plots, with the median of each group in the center line, the box drawn from the first to the third quartile and whiskers representing the minimum and maximum of each group. A Shapiro–Wilk test confirmed that all data were normally distributed. Significant differences are marked with * (*p* < 0.05).

**Figure 8:**
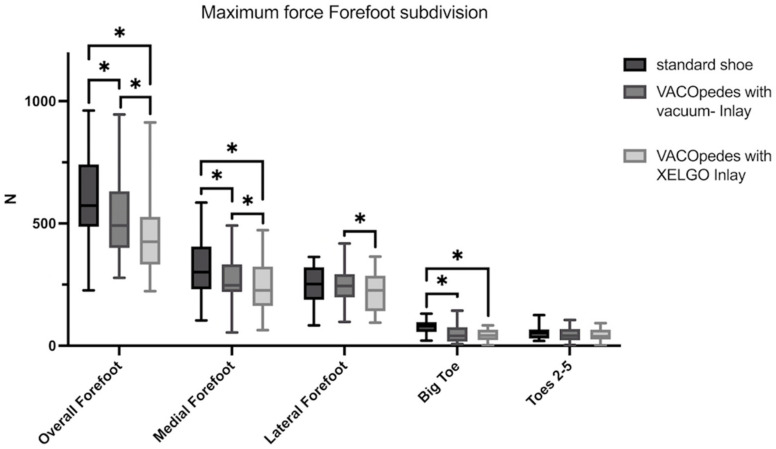
Maximum force of the forefoot in Newton (N) for each group, separated into overall, medial forefoot, lateral forefoot, big toe and toes 2–5. Data are presented in box plots, with the median of each group in the center line, the box drawn from the first to the third quartile and whiskers representing the minimum and maximum of each group. A Shapiro–Wilk test confirmed that all data were normally distributed. Significant differences are marked with * (*p* < 0.05).

In the medial forefoot area, both inlays lowered the peak pressure and maximum force compared to the OTS significantly (Figure 7 and Figure 8, Table 2). Further, there was a significantly lower peak pressure (*p* = 0.002, Figure 7) and maximum force (*p* = 0.012, Figure 8) in the VACOpedes^®^ orthosis with the XELGO^®^ inlay compared to the vacuum inlay.

**Figure 9:**
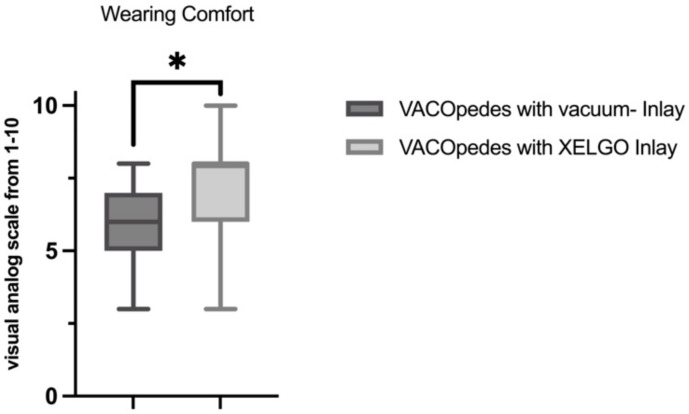
Wearing comfort for the VACOpedes with vacuum-inlay and XELGO inlay according to a visual analog scale ranging from 1–10, with 10 representing the best wearing comfort. A Shapiro–Wilk test confirmed that all data were normally distributed. Significant differences are marked with * (*p* < 0.05).

Regarding the wearing comfort, subjects rated the VACOpedes^®^ orthosis with a XELGO^®^ inlay as significantly more comfortable than the VACOpedes^®^ orthosis with a vacuum inlay (vacuum inlay 5.68 ± 1.46, XELGO^®^ inlay 7.24 ± 1.50, *p* < 0.001, Figure 9).

## 4. Discussion

This is the first study to evaluate a novel self-adapting inlay in a widespread foot orthosis compared to a vacuum-based inlay and a standard shoe. In our study, the VACOpedes^®^ foot orthosis with XELGO^®^ inlay appears to reduce plantar pressure equally to the vacuum inlay in most areas of the foot, while there is a significant difference in the favor of the XELGO^®^ inlay in the medial forefoot. In addition, the XELGO^®^ inlay is more comfortable to wear and is easier to use as there is no need for vacuum suction.

XELGO^®^ is a recent development by OPED GmbH, so this is the first study evaluating the new technology regarding plantar pressure, maximum force and wearing comfort. Thus, the comparability of our study is limited. However, previous studies have evaluated the effect on plantar pressure of vacuum technology in a foot orthosis compared to a standard shoe. Nagel et al. (2009) showed a significant reduction in peak plantar pressure with the VACOpedes^®^ orthosis compared to a standard shoe in the hindfoot (307 vs. 194 kPa) and forefoot (421 vs. 192 kPa), whereas no effect was observed in the midfoot (154 vs. 147 kPa) [14]. Ehrnthaller et al. also demonstrated in an analysis of five different orthoses a reduced peak plantar pressure with a short walker boot compared to a standard shoe in the hindfoot (195 vs. 174 kPa) and the forefoot (248 vs. 155 kPa), in contrast to an increase in the midfoot (77 vs. 109 kPa) [15]. These results are in line with our findings of a predominantly forefoot unloading effect of this foot orthosis. However, we observed a non-significant trend towards a simultaneous unloading of the midfoot of 18%. Ideally, research on footwear and orthoses should consider both subjective comfort and objective biomechanical effects. Puszczalowska-Lizis et al. demonstrated that perceptions of footwear comfort vary by sex, with women generally reporting higher comfort levels than men. A better perception of comfort is linked to a lower risk of falling in people aged 65–74. Future studies could investigate how different orthoses affect both plantar pressure and the perceived comfort of footwear in older adults, bridging the gap between these topics [16,17].

The materials of the insoles of different foot orthoses showed different effects on plantar pressure during walking, e.g., polyurethane, polyethylene and ethyl vinyl acetate provided a greater reduction in plantar pressure compared to carbon graphite [18]. Simonds et al. investigated different types of granular jamming materials, including rice, poppy seeds, micropolystyrene, and polystyrene beads with vacuum suction and demonstrated an excellent energy absorption of all granular materials and an increased stiffness with increasing vacuum pressure [19]. With XELGO^®^, the efficient use of polystyrene beads in combination with vacuum for the reduction of plantar pressure is further developed. Here, the same polystyrene beads as in the vacuum-based inlay are moistened with a highly viscous liquid and bind together under pressure. This composition allows for a continuous adaption to the foot anatomy as well as the reversible effect by kneading and flattening the inlay without the need for vacuum suction [19]. It is well known that changes in plantar pressures occur due to changes of the ankle position within the orthosis. Even small changes in ankle position in dorsiflexion or plantarflexion have a significant effect on the resulting forefoot and hindfoot plantar pressures when walking in a prefabricated boot [20]. In our study design, the two boots used allowed full range of motion of the ankle. In addition, XELGO^®^ includes a textile membrane, which ensures breathability and ventilation to prevent heat build-up. This combination self-adapting inlay and the textile membrane might be reason for higher preserved wearing comfort.

The XELGO^®^ inlay has an advantage over vacuum insoles because it provides the same level of relief to the foot, increases comfort, simplifies handling, and might reduce the risk of soft tissue damage or falls due to the inadequate support of the orthosis, especially in the geriatric patient population. Because of the predominant unloading of the forefoot, the VACOpedes^®^ orthosis with a XELGO^®^ inlay could be used not only in traumatology and orthopedics but also in the treatment of chronic ulcers, especially in diabetic patients [10,11,21,22,23].

In patients with diabetic foot ulcers, the VACOpedes orthosis was preferred by many patients and appears to be as effective as other removable cast walkers [23]. A recently published study using a combination of custom rocker and self-adjusting insoles in patients with diabetes mellitus and loss of protective sensation successfully reduced peak pressure. Insoles reduced peak plantar pressure (<200 kPa) in the toes and central and lateral forefoot, but not in the heels.

This study has some limitations. We conducted the study in 25 healthy subjects. A larger cohort might have improved the significance of our study, but according to the a priori power analysis, our study is not underpowered. The exclusion of patients with foot and ankle pathologies limits the translation of the results of our study to the clinic. However, we decided to reduce the confounding variables that would have influenced the plantar pressure and thus improve the comparability of our study. Despite there being evidence of an enhanced perception of wearing comfort in women, we did not analyze any gender-specific differences. Due to the design of the study, the orthoses were applied by the same person who was acquainted with the system, but subjects were not blinded. This could introduce a confirmation bias into the wearing comfort analysis.

## 5. Conclusions

The VACOpedes^®^ orthosis with a new XELGO^®^ inlay showed at least equivalent relief in all pressure distribution measurements analyzed, as well as greater relief and unloading in the forefoot area, especially in the first ray, than the VACOpedes^®^ orthosis with a vacuum inlay, as well as increased wearing comfort. Future research and clinical applications of the novel XELGO^®^ technology in foot orthoses and ankle foot orthoses must confirm these promising results by investigating its application to foot and ankle pathologies as well as soft tissue-related problems.

## Figures and Tables

**Table 1:** Peak pressure in kiloPascal (kPa) for each area and all group comparisons. OTS = Off-the-shelf shoe. A Shapiro–Wilk test confirmed that all data were normally distributed. Significant differences are marked in bold with *p* < 0.05.

Foot Region	Comparison	Mean ± SD First Comparator	Mean ± SD Second Comparator	*p*-Value
Hindfoot	OTS vs. VACOpedes with vacuum inlay	235.5 ± 40.5	205.5 ± 48.4	**0.003**
	OTS vs. VACOpedes with XELGO inlay	235.5 ± 40.5	197.8 ± 37.6	**<0.001**
	VACOpedes with vacuum inlay vs. VACOpedes with XELGO inlay	205.5 ± 48.4	197.8 ± 37.6	0.704
Midfoot	OTS vs. VACOpedes with vacuum inlay	147.9 ± 58.5	123.4 ± 40.7	**0.007**
	OTS vs. VACOpedes with XELGO inlay	147.9 ± 58.5	121.3 ± 38.8	**0.009**
	VACOpedes with vacuum- inlay vs. VACOpedes with XELGO inlay	123.4 ± 40.7	121.3 ± 38.8	0.919
Forefoot	OTS vs. VACOpedes with vacuum inlay	262.9 ± 58.4	202.1 ± 45.5	**<0.001**
	OTS vs. VACOpedes with XELGO inlay	262.9 ± 58.4	171 ± 46.9	**<0.001**
	VACOpedes with vacuum inlay vs. VACOpedes with XELGO inlay	202.1 ± 45.5	171 ± 46.9	**<0.001**

**Table 2:** Peak pressure in kiloPascal (kPa) for each forefoot area and all group comparisons. OTS = Off-the-shelf shoe. A Shapiro–Wilk test confirmed that all data were normally distributed. Significant differences are marked in bold with *p* < 0.05.

Foot Region	Comparison	Mean ± SD First Comparator	Mean ± SD Second Comparator	*p*-Value
Overall Forefoot	OTS vs. VACOpedes with vacuum inlay	264.1 ± 12.1	201 ± 9.4	**<0.001**
	OTS vs. VACOpedes with XELGO inlay	264.1 ± 12.1	171 ± 9.6	**<0.001**
	VACOpedes with vacuum inlay vs. VACOpedes with XELGO inlay	201 ± 9.4	171 ± 9.6	**<0.001**
Medial Forefoot	OTS vs. VACOpedes with vacuum inlay	244.4 ± 13.8	181.7 ± 9.1	**<0.001**
	OTS vs. VACOpedes with XELGO inlay	244.4 ± 13.8	158.6 ± 10.3	**<0.001**
	VACOpedes with vacuum inlay vs. VACOpedes with XELGO inlay	181.7 ± 9.1	158.6 ± 10.3	**0.002**
Lateral Forefoot	OTS vs. VACOpedes with vacuum inlay	201.5 ± 11.4	161.3 ± 9.5	**<0.001**
	OTS vs. VACOpedes with XELGO inlay	201.5 ± 11.4	153.3 ± 9.3	**<0.001**
	VACOpedes with vacuum inlay vs. VACOpedes with XELGO inlay	161.3 ± 9.5	153.3 ± 9.3	0.108
Big Toe	OTS vs. VACOpedes with vacuum inlay	213.7 ± 13.1	137.7 ± 16.6	**<0.001**
	OTS vs. VACOpedes with XELGO inlay	213.7 ± 13.1	116.7 ± 12.1	**<0.001**
	VACOpedes with vacuum inlay vs. VACOpedes with XELGO inlay	137.7 ± 16.6	116.7 ± 12.1	0.088
Toes 2–5	OTS vs. VACOpedes with vacuum inlay	102.5 ± 7.2	96.1 ± 10.4	0.542
	OTS vs. VACOpedes with XELGO inlay	102.5 ± 7.2	85.8 ± 8.9	**0.023**
	VACOpedes with vacuum inlay vs. VACOpedes with XELGO inlay	96.1 ± 10.4	85.8 ± 8.9	0.233

## Data Availability

The raw data supporting the conclusions of this article will be made available by the authors on request.

## Abbreviations

The following abbreviations are used in this manuscript:

kPa	kiloPascal
N	Newton
OTS	Off-the-shelf shoe
